# Analysis of Hypoxia and Hypoxia-Like States through Metabolite Profiling

**DOI:** 10.1371/journal.pone.0024741

**Published:** 2011-09-12

**Authors:** Julie E. Gleason, David J. Corrigan, James E. Cox, Amit R. Reddi, Lauren A. McGinnis, Valeria C. Culotta

**Affiliations:** 1 Department of Biochemistry and Molecular Biology, Johns Hopkins University Bloomberg School of Public Health, Baltimore, Maryland, United States of America; 2 Metabolomics Core Research Facility, University of Utah School of Medicine, Salt Lake City, Utah, United States of America; Newcastle University, United Kingdom

## Abstract

**Background:**

In diverse organisms, adaptation to low oxygen (hypoxia) is mediated through complex gene expression changes that can, in part, be mimicked by exposure to metals such as cobalt. Although much is known about the transcriptional response to hypoxia and cobalt, little is known about the all-important cell metabolism effects that trigger these responses.

**Methods and Findings:**

Herein we use a low molecular weight metabolome profiling approach to identify classes of metabolites in yeast cells that are altered as a consequence of hypoxia or cobalt exposures. Key findings on metabolites were followed-up by measuring expression of relevant proteins and enzyme activities. We find that both hypoxia and cobalt result in a loss of essential sterols and unsaturated fatty acids, but the basis for these changes are disparate. While hypoxia can affect a variety of enzymatic steps requiring oxygen and heme, cobalt specifically interferes with diiron-oxo enzymatic steps for sterol synthesis and fatty acid desaturation. In addition to diiron-oxo enzymes, cobalt but not hypoxia results in loss of labile 4Fe-4S dehydratases in the mitochondria, but has no effect on homologous 4Fe-4S dehydratases in the cytosol. Most striking, hypoxia but not cobalt affected cellular pools of amino acids. Amino acids such as aromatics were elevated whereas leucine and methionine, essential to the strain used here, dramatically decreased due to hypoxia induced down-regulation of amino acid permeases.

**Conclusions:**

These studies underscore the notion that cobalt targets a specific class of iron proteins and provide the first evidence for hypoxia effects on amino acid regulation. This research illustrates the power of metabolite profiling for uncovering new adaptations to environmental stress.

## Introduction

Oxygen sensing and homeostasis is a complex process and can represent the driving force for tissue differentiation and disease progression. In multicellular eukaryotes, adaptation to low oxygen is mediated in part through a complex reprogramming of gene expression via the action of the HIF-1α trans-regulator. The HIF-1α polypeptide is stabilized by hypoxia and induces expression of genes for vascularization, erythropoiesis, tissue oxygenation and energy metabolism [1]. HIF-1α itself is regulated by a family of proline and arginine hydroxylases that sense and respond to oxygen by hydroxylating HIF-1α and triggering its degradation through the proteosome. These hydroxylases are non-heme iron enzymes that require oxygen, 2-oxoglutarate and ascorbate [2], [3].

In widespread laboratory studies of hypoxic gene regulation, cobalt is often used to produce a “hypoxia-like” state (for examples, see [4], [5], [6], [7], [8].) As with low oxygen, cobalt can stabilize HIF-1α through inhibition of its hydroxylation by the aforementioned prolyl and arginyl hydroxylases. Cobalt may disrupt the iron co-factor of the hydroxylases and may also lead to loss of the ascorbate needed for hydroxylation [9], [10], [11].

In organisms that do not produce HIF-1α, cobalt can still elicit a hypoxia-like response. In the case of the fungi *C. neoformans* and *S. pombe,* cobalt can induce the Sre1-dependent gene expression system for sterol synthesis in a manner akin to hypoxia [12]. Cobalt can also induce a hypoxia-like gene response in the bakers’ yeast *S. cerevisiae* that lack both HIF-1α and Sre1. In this case, hypoxia and cobalt overlap in regulating genes involved in respiration and fatty acid biosynthesis [13], [14], [15]. The mechanism for this hypoxia-like effect of cobalt is not well understood. In addition to partially mimicking hypoxia, cobalt can induce an iron starvation-like response. Specifically, cobalt induces the *S. cerevisiae* Aft1p trans regulator for induction of iron uptake and metabolism genes [16]. However, hypoxia does not induce Aft1p and if anything genes for high affinity iron uptake are repressed with low oxygen [17]. Aft1p is believed to sense and respond to loss of mitochondrial Fe-S biogenesis [18], [19], yet how cobalt links into Aft1p sensing is not understood. In any case, these observations illustrate the complexity of metal toxicity. The gene reprogramming effects of cobalt can extend well beyond induction of a hypoxia-like state.

In the studies described herein, we have applied a low molecular weight metabolome profiling approach in *S. cerevisiae* to gain a more complete picture of cellular hypoxia versus the “hypoxia-like state” induced by cobalt. Such analysis of individual metabolites allows us to directly view impacts on cell metabolism that may be missed through traditional gene or protein expression profiles. We report here that the hypoxia-simulation properties of cobalt in yeast include a reduction in cellular sterols and unsaturated fatty acids (UFAs). The underlying mechanism involves cobalt-mediated inhibition of enzymatic steps involving diiron-oxo enzymes. Yet the impact of hypoxia and cobalt on metabolism diverged beyond this point. Cobalt, but not hypoxia, also targeted steps involving 4Fe-4S dehydratase enzymes of the mitochondria. Hypoxia, but not cobalt, was associated with disturbances in amino acid pools and amino acid transport. These studies exemplify the power of metabolite profiling in revealing adaptive responses to environmental change.

## Results

To comparatively analyze the effects of cobalt and hypoxia on cell metabolism, we examined a pool of metabolites by gas chromatography/mass spectrometry (GC/MS). Included in the list were select compounds of the TCA cycle, certain amino acids and their precursors, metabolites of sterol biosynthesis and several others (summarized in Table S1). In separate studies fatty acids were monitored by gas chromatography and additional amino acids were analyzed by ion exchange chromatography. All metabolites were analyzed from *S. cerevisiae* cells that were allowed to double three times in a minimal medium either in air untreated, in air treated with 2 mM cobalt or in an anaerobic environment to create a hypoxic condition. Three doublings represents the maximal growth that could be achieved under anaerobic conditions without supplementation of exogenous sterols and unsaturated fatty acids. In review of the findings, we observed some particularly notable effects on metabolites related to sterols and lipids, Fe-S enzymes and amino acids.

### Cobalt versus hypoxia effects on sterol biosynthesis

**Figure 1:**
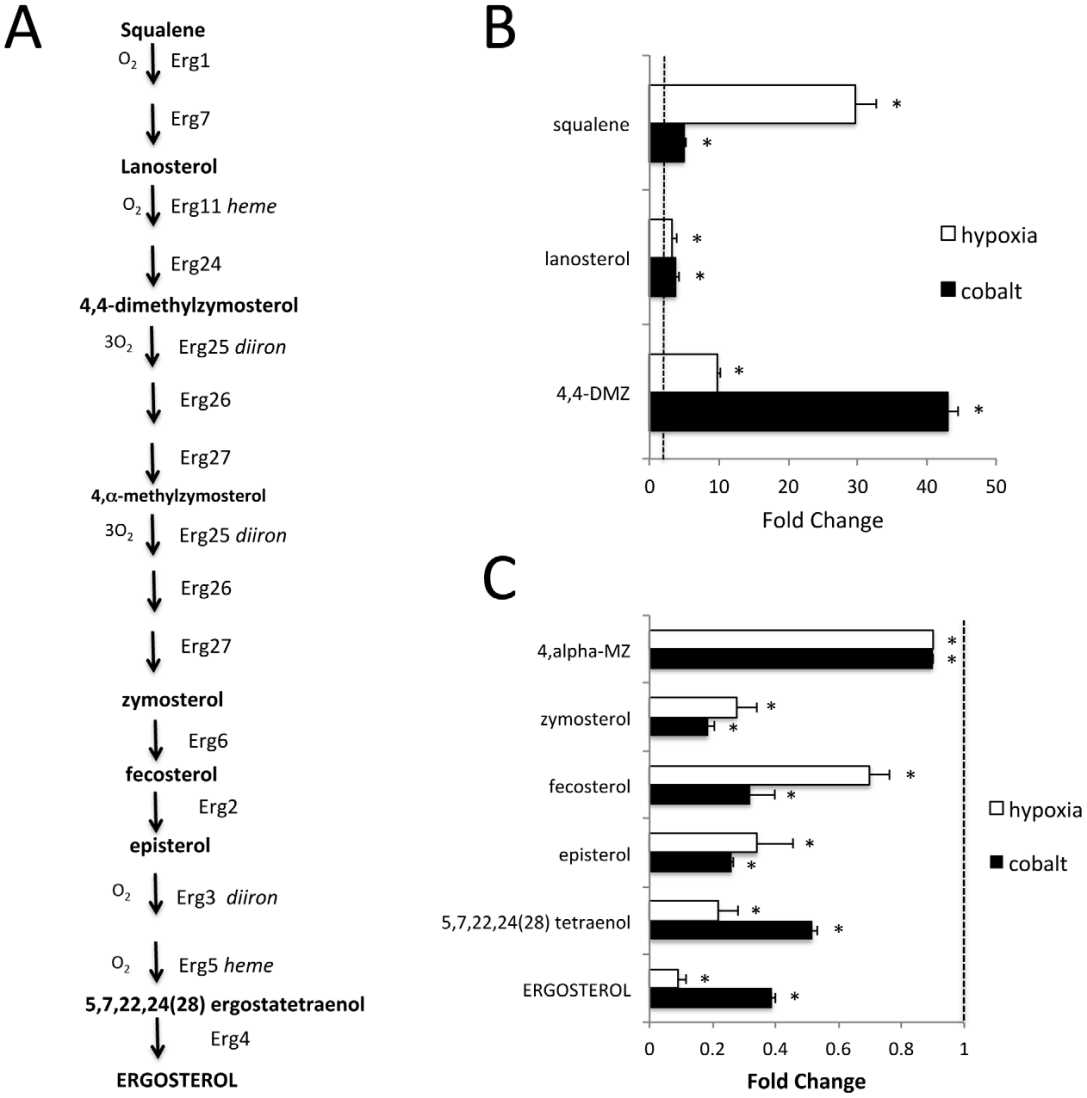
Effects of cobalt and hypoxia on sterol biosynthesis. A) Schematic illustrating the sterol biosynthetic pathway. The iron (*heme* and *diiron*) and oxygen (O_2_) requiring steps are indicated. B,C) Cells grown in minimal medium as described in Materials and Methods were analyzed for metabolites corresponding to upstream (B) and downstream (C) portions of the sterol biosynthetic pathway. White and black bars equal hypoxia and cobalt treated samples respectively. Results represent fold changes over control (aerobically grown with no cobalt) and are averages of 8 samples; error bars  =  standard deviation. Statistically significant differences over control (P value  = 0.05) are designated with asterisks. Dotted line  = 1.0 value assigned to control. DMZ and MZ represent dimethylzymosterol and methylzymosterol.

*S. cerevisiae* and related yeast produce ergosterol rather than cholesterol for incorporation into membranes. Like cholesterol, the biosynthesis of ergosterol involves multiple oxygen-requiring steps (Fig. 1A) and as expected, ergosterol levels drop an order of magnitude with hypoxia (Fig. 1C). Ergosterol levels are also lowered with cobalt, with cells grown in minimal medium (Fig. 1C) or enriched YPD medium (Fig. S1B). The metabolites immediately upstream of ergosterol, up to and including zymosterol, are also decreased with both hypoxia and cobalt (Fig. 1C). By comparison, in the far upstream portions of the pathway (squalene to 4,4-dimethlyzymosterol) the precursors tend to increase rather than decrease with both cobalt and hypoxia (Fig. 1B). Hypoxia has a most dramatic effect (30 fold increase) on squalene, representing the substrate for Erg1p, the first oxygen-requiring step in ergosterol biosynthesis. Cobalt effects were most pronounced (over 40 fold increase) with 4,4-dimethylzymosterol, the substrate for Erg25p representing the first diiron-oxo enzymatic step in the pathway (Fig. 1A–B), suggesting that Erg25p is inhibited by cobalt. Hypoxia also increased levels of 4,4-dimethylzymosterol (Fig. 1B).

**Figure 2:**
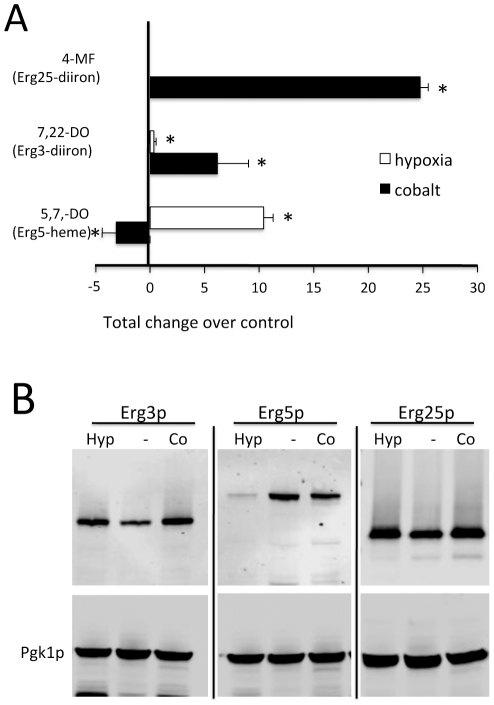
Effects of cobalt and hypoxia on sterol biosynthetic enzymes Erg3p, Erg5p and Erg25p. Cells grown in minimal medium as in Fig. 1 were analyzed for sterol metabolites and expression of sterol biosynthetic enzymes. A) Byproducts from blocks in enzymatic steps for *S. cerevisiae* Erg25p, Erg3p and Erg5p are indicated. The arbitrary units shown represent the quantity of metabolite detected by GC/MS above that of control untreated samples which  = 0 (undetectable) in the case of 4 methyl fecosterol (4-MF) and ergosta 7,22-dien-3B-ol (7,22-DO). Control untreated samples had a small level of ergosta 5,7-dien-ol (5,7-DO) which was increased with hypoxia, but decreased with cobalt treatment as depicted by the negative value. B) Cells expressing TAP tagged versions of the indicated proteins were analyzed by immunoblot using an antibody directed against TAP. Blots were also probed with anti-PGK1 as a control. Shown are expression levels of TAP tagged-Erg3p (63.7 kDa), Erg5p (82.3 kDa) and Erg25p (57.5 kDa). “Hyp”, hypoxia treated; “−“, control; “Co”, cobalt. Results are representative of two (Erg25p) and three (Erg3p and Erg5p) experimental trials.

It is known that specific blockages in ergosterol biosynthesis can lead to hyperaccumulation of key byproducts that are not generally accumulated in yeasts. For example, loss of Erg25p can lead to hyperaccumulation of 4-methyl fecosterol (4-MF) [12]. Although 4-MF was not detected in control untreated cells, it accumulated to robust levels in cobalt-treated cells grown either in minimal (Fig. 2A) or in enriched YPD medium (Fig. S1C) consistent with inactivation of the diiron-oxo Erg25p enzyme. A second diiron enzyme in ergosterol biosynthesis is Erg3p and a byproduct of Erg3p inactivation is ergosta-7,22-dien-3B-ol (7,22-DO) [20]. 7,22 DO was absent from control cells, but accumulated with cobalt treatment (Fig. 2A). Overall, the marked accumulation of 7,22 DO and 4-MF strongly indicates that cobalt inactivates the diiron enzymes Erg3p and Erg25p involved in ergosterol biosynthesis. A similar effect of cobalt on diiron containing enzymes for sterol biosynthesis has been reported for *Cryptococcus neoformans* [12]. 7,22 DO and 4-MF did not hyperaccumulate with hypoxia, presumably due to the oxygen requirement for biosynthesis of these byproducts.

Another specific marker for sterol defects is ergosta-5,7-dienol (5,7-DO), a hallmark for blockages in Erg5p [21]. Erg5p represents the terminal heme and oxygen-requiring step in ergosterol biosynthesis (Fig. 1A). If anything, 5,7-DO levels dropped with cobalt treatment (Fig. 2A) similar to cobalt effects on other metabolites in this downstream portion of the ergosterol pathway (Fig. 1). Although cobalt shows no evidence for Erg5p inactivation, hypoxia was associated with an increase in 5,7-DO consistent with a loss in activity of this heme and oxygen requiring enzyme (Fig. 2A).

Prompted by the effects of cobalt and hypoxia on Erg3p, Erg5p and Erg25p steps, we examined levels of the corresponding TAP tagged versions of the polypeptides. As seen in Fig. 2B, there was no loss in Erg3 and Erg25 polypeptides with cobalt. If anything, Erg3p and Erg25p levels increased somewhat with cobalt similar to effects of hypoxia. *ERG3* and *ERG25* (but not *ERG5*) genes are known to be induced in response to low sterol levels during hypoxia [22] and the same may hold true for cobalt treated cells. Unlike Erg3p and Erg25p, Erg5p levels substantially decreased with hypoxia (Fig. 2B). Erg5p requires heme, and since heme biosynthesis is oxygen-dependent, loss of the heme co-factor during hypoxia might trigger turnover of the Erg5 polypeptide. Thus while both cobalt and hypoxia result in a loss of ergosterol, they do so by varying mechanisms. Hypoxia can affect multiple oxygen-requiring steps particularly the first step involving Erg1p as well as Erg3p, Erg5p, Erg11p and Erg25p, whereas cobalt specifically targets steps involving the two diiron-oxo enzymes Erg3p and Erg25p.

### Parallel effects of cobalt and hypoxia on fatty acids

**Figure 3:**
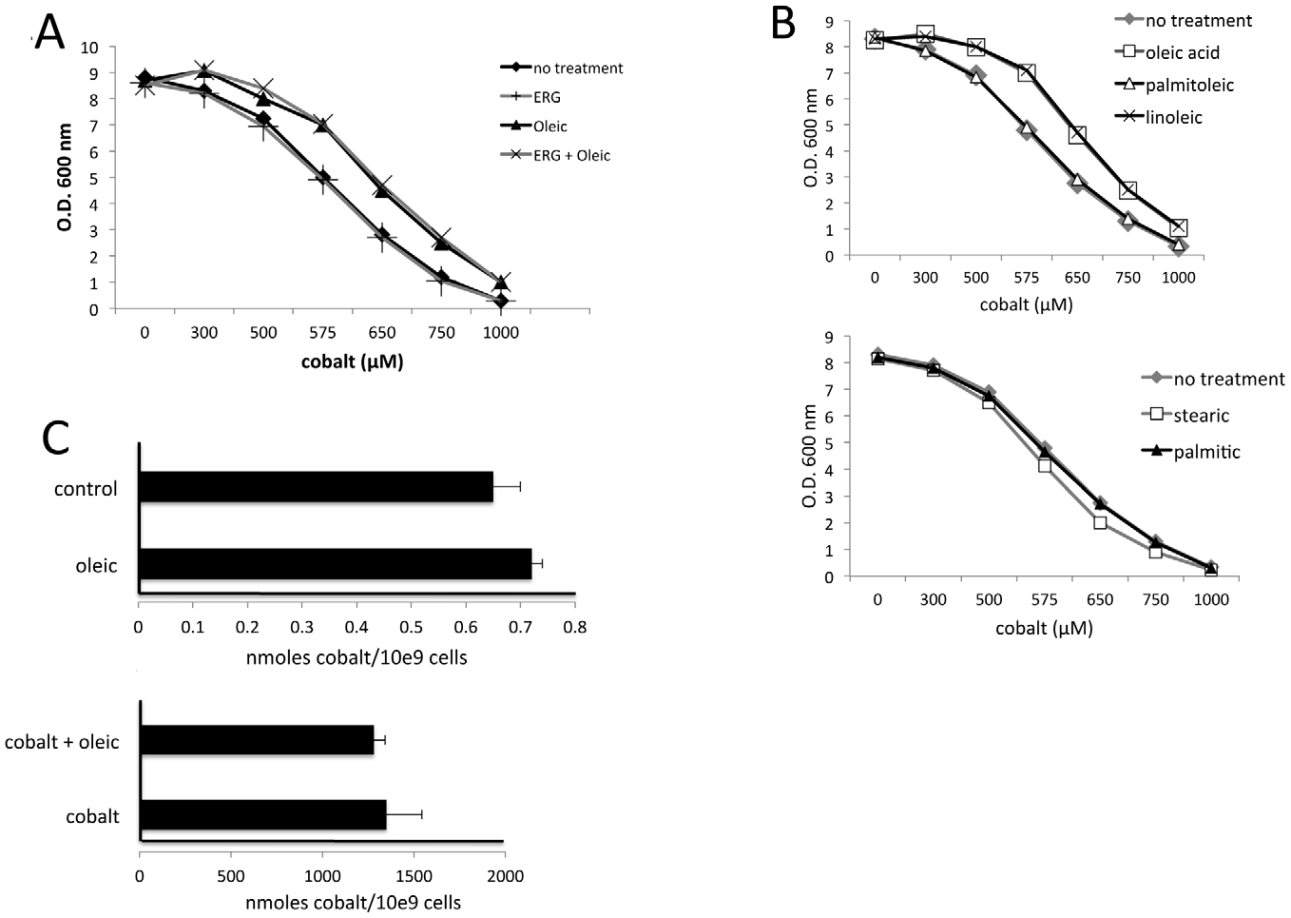
Effects of lipids on cobalt toxicity. A,B) Cells were grown for 18 hours in enriched YPD medium in the presence of the indicated concentrations of CoCl_2_ also supplemented where indicated with (A) 15 mg/L ergosterol and/or 0.5 mM Oleic acid; and (B) 0.5 mM concentrations of the indicated unsaturated (oleic, palmitoleic and linoleic acid) or saturated (stearic and palmitic acid) fatty acids. Total growth following 18 hours was measured at an optical density at 600 nM. Results represent the averages of three independent cultures where error ≤2% in all cases. C) Cells grown as in Fig. 3A in the presence of 600 µM CoCl_2_ and/or 0.5 mM oleic acid were subjected to cobalt analysis by atomic absorption spectroscopy. Results represent the averages of three independent cultures and error bars represent the total range.

Although cobalt treated cells have low ergosterol in both minimal medium (Fig. 1C) and in enriched medium (Fig. S1B), ergosterol supplementation did not improve growth of cobalt treated cells (Fig. 3A). However, we consistently observed some enhancement of growth in cobalt by addition of unsaturated fatty acids (UFAs). Specifically, the concentration of cobalt required to inhibit growth by 50% was raised from ≈600 µM to ≈675 µM by supplementing enriched medium with either the 18:1 UFA oleic acid or the 18:2 n-6 UFA linoleic acid (Fig. 3A, 3B). These effects on cobalt toxicity were not due to changes in cellular uptake of the metal; the accumulation of cobalt was unchanged by oleic acid treatments as determined by atomic absorption spectroscopy (Fig. 3C). We therefore predicted that the 18 carbon UFAs were reversing a downstream product of cobalt toxicity.

**Figure 4:**
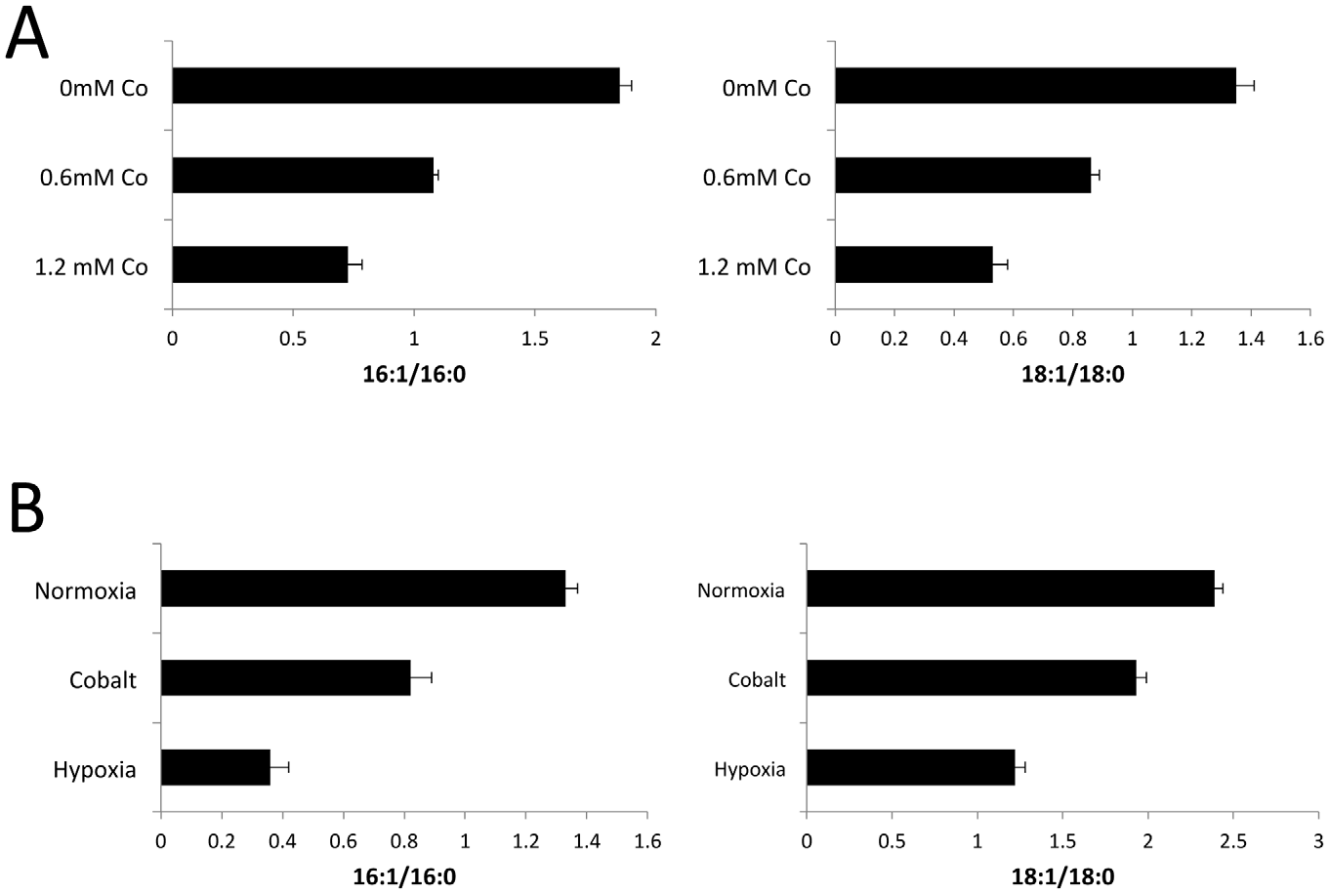
Gas chromatography analysis of fatty acids. Cells were grown either in (A) enriched medium as in Fig. 3 or in (B) minimal medium as in Fig. 1. Fatty acids were extracted and analyzed by gas chromatography as described in Materials and Methods. Results are presented as the ratio of unsaturated/saturated fatty acids for 16 and 18 carbon lipids as indicated and represent the averages of 2–3 independent cultures, error bars represent range.

Fatty acid desaturation in *S. cerevisiae* is accomplished by a diiron-oxo desaturase known as Ole1p. Ole1p converts the 16:0 and 18:0 saturated fatty acids (SFAs) palmitic and stearic to the 16:1 and 18:1 UFAs, palmitoleic and oleic acids [23], [24]. An index of Ole1p activity *in vivo* is the cellular ratio of UFAs/SFAs which is maintained at 1.5 – 2.5 in standard growth medium [23] (Fig. 4). This ratio of UFA/SFA was consistently lowered in cobalt treated cells grown either in enriched (Fig. 4A) or minimal (Fig. 4B) medium. Hypoxia also reduced this ratio as would be expected based on the oxygen requirement for Ole1p (Fig. 4B).

**Figure 5:**
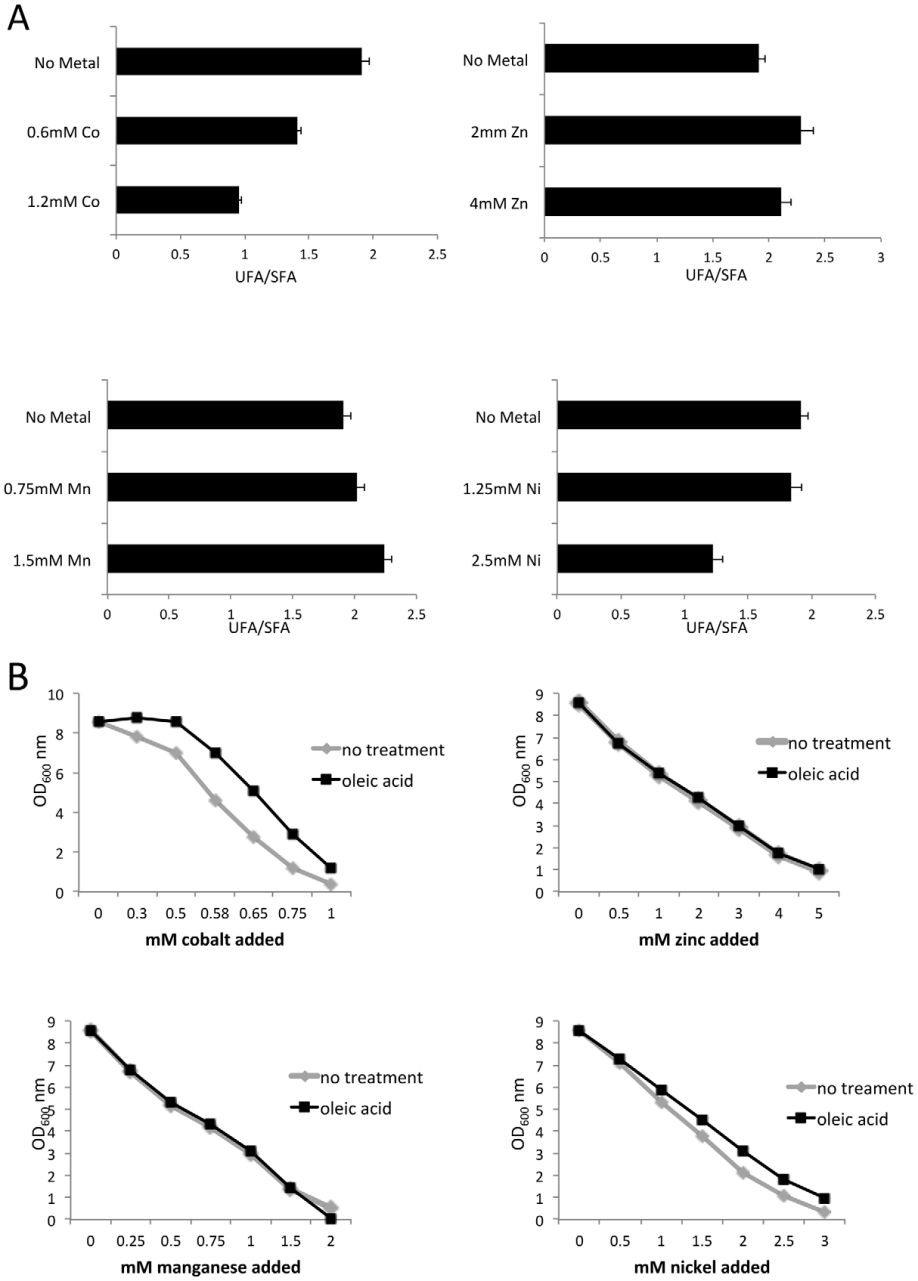
Inhibition of lipid desaturation is specific to cobalt. Cells were grown in enriched medium as in Fig. 3 in the presence of the designated concentrations of CoCl_2_, ZnCl_2_, MnCl_2_ or NiCl_2_. A) Lipid content determined as in Fig. 4. Total lipid desaturation (16+18 carbon) is determined as a ratio of unsaturated oleic plus palmitoleic/saturated stearate plus palmitate (UFA/SFA). The concentration of metals chosen were equivalent to levels that inhibit 18 hour growth by 50% and a concentration twice this value. B) Toxicity of metals in the presence or absence of 0.5 mM oleic acid was determined as in Fig. 3.

We tested whether this effect of cobalt on fatty acids represented a general metal toxicity phenomenon. In the experiment of Fig. 5, cells were treated with concentrations of zinc (2 mM), manganese (0.75 mM) and nickel (1.25 mM) that inhibited growth in enriched medium to the same extent as 0.6 mM cobalt and also at twice these doses. The UFA/SFA ratio was unchanged with zinc and manganese and only lowered by exceedingly toxic doses of nickel (Fig. 5A). As expected, oleic acid had little or no impact on toxicity of these metals (Fig. 5B). Of the metals tested, cobalt appeared most effective in disrupting fatty acid desaturation.

**Figure 6:**
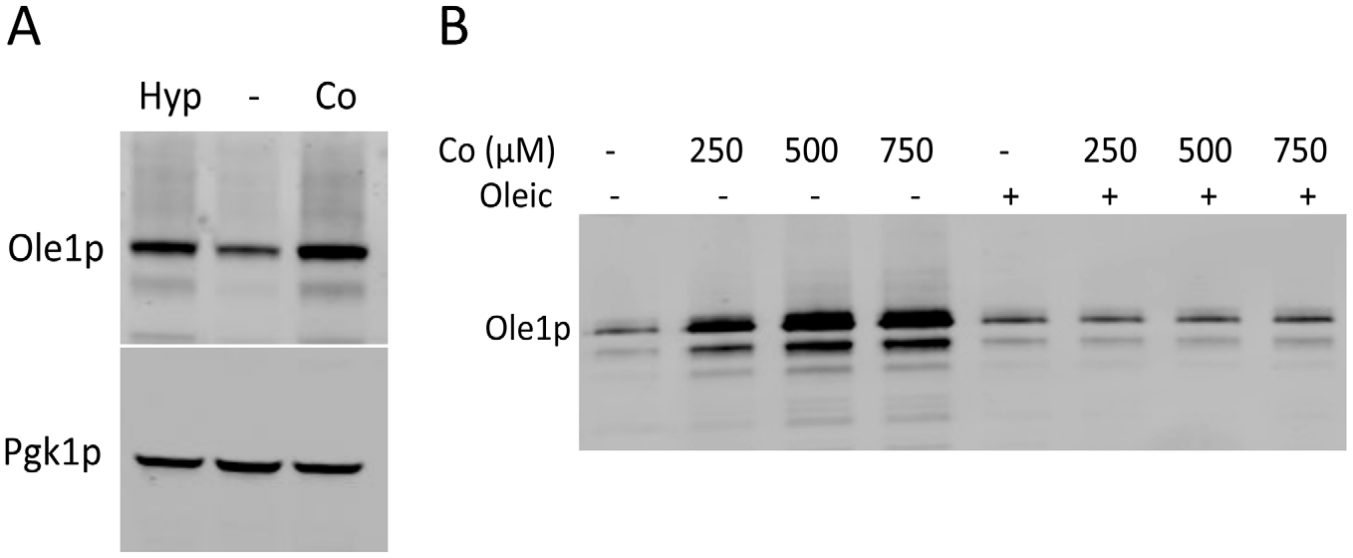
Effects of cobalt and hypoxia on Ole1p. Immunoblot analysis was conducted as in Fig. 2B of cells expressing the TAP tagged version of Ole1p grown either in (A) minimal medium as in Fig. 1 or in (B) enriched medium as in Fig. 3 and supplemented with the indicated concentrations of CoCl_2_. Oleic: +, cells supplemented with 0.5 mM oleic acid. Results are representative of two (part A) or three (part B) experimental trials.

We examined levels of the Ole1p desaturase in cobalt treated cells. TAP tagged Ole1p increased with cobalt similar to the effects of hypoxia (Fig. 6A), consistent with the known transcriptional induction of *OLE1* by cobalt and hypoxia [13], [14], [25]. Supplementing cells with exogenous oleic acid completely reversed the cobalt induction of Ole1p (Fig. 6B). Hence, the reduction in UFAs with cobalt appears responsible for the hypoxia-mimic effect of cobalt in inducing *OLE1.* We conclude from these studies on UFAs that like hypoxia, cobalt inhibits lipid desaturation by Ole1p. Together with findings on sterols (Figs. 1,2), the class of diiron-oxo enzymes including Ole1p, Erg3p and Erg25p represent potential targets of cobalt toxicity.

### Cobalt inhibition of Fe-S dehydratase enzymes

**Figure 7:**
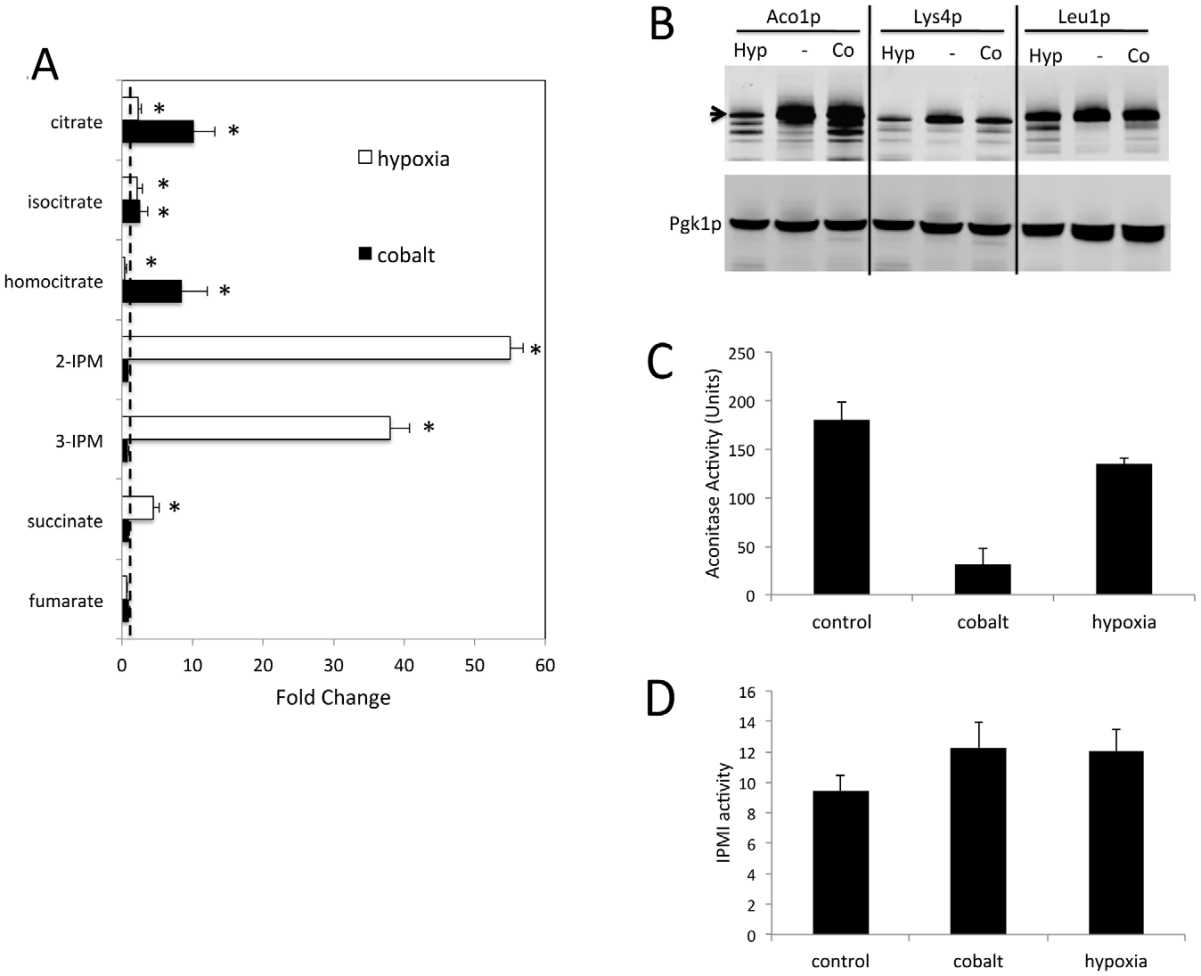
Effects of cobalt on Fe-S enzymes. Cells were grown in minimal medium as in Fig. 1 and were assayed for (A) designated metabolites by GC/MS; (B,C) protein levels and enzymatic activity of indicated Fe-S proteins. A) Levels of select Fe-S enzyme substrates and products presented as fold change over control untreated samples as in Fig. 1B,C. Statistically significant differences over control (P value ≤0.05) are designated with asterisks. Dotted line  = 1.0 value assigned to control. Citrate and isocitrate - substrate and product of Aconitase; homocitrate - homoaconitase substrate; 2-IPM and 3-IPM (isopropyl malate) - substrate and product of IPMI (Leu1p); succinate and fumarate - substrate and product of succinate dehydrogenase. B) Immunoblot analysis of cells expressing TAP tagged versions of Aco1p (aconitase 106 kDa), Lys4p (homoaconitase 96 kDa) and Leu1p (IPMI 106.5 kDa). Results are representative of three (Leu1p), four (Lys4p) and five (Aco1p) experimental trials. C–D) Cell lysates prepared and assayed for aconitase (C) and IPMI (D) activity as described in Materials and Methods. 1 unit of activity is defined as 1 nmole of substrate consumed per min per mg of protein.

In addition to affecting diiron-oxo enzymes, cobalt-treated cells exhibited specific deficiencies in Fe-S enzymes. By GC/MS, there was a dramatic increase (≈10 fold) in citrate and homocitrate, substrates for the Fe-S enzymes aconitase and homoaconitase (Fig. 7A). Aconitase and homoaconitase are members of the 4Fe-4S dehydratase enzymes [26]. Another highly homologous member of this family includes isopropyl malate isomerase (IPMI also known as Leu1p), representing the second step in leucine biosynthesis. However, the 2-IPM substrate for IPMI was not significantly affected by cobalt treatment. We also observed no change in the substrate for an unrelated Fe-S enzyme, succinate dehydrogenase (Fig. 7A). It appears that the aconitase and homoaconitase steps are particularly vulnerable to cobalt. To further investigate this, we examined the polypeptide levels and enzymatic activities of the 4Fe-4S dehydratase enzymes. By immunoblot, there was no appreciable effect of cobalt on the polypeptides levels of TAP-tagged aconitase (Aco1p), homoaconitase (Lys4p) or IPMI (Leu1p) (Fig. 7B). However, cobalt treatment correlated with a dramatic drop in aconitase enzyme activity, while IPMI activity was not affected (Fig. 7C,D). These results on enzyme activity completely track with our metabolite profile (Fig. 7A) where cobalt leads to a rise in the citrate substrate for aconitase but not the 2-IPM substrate for IPMI.

**Figure 8:**
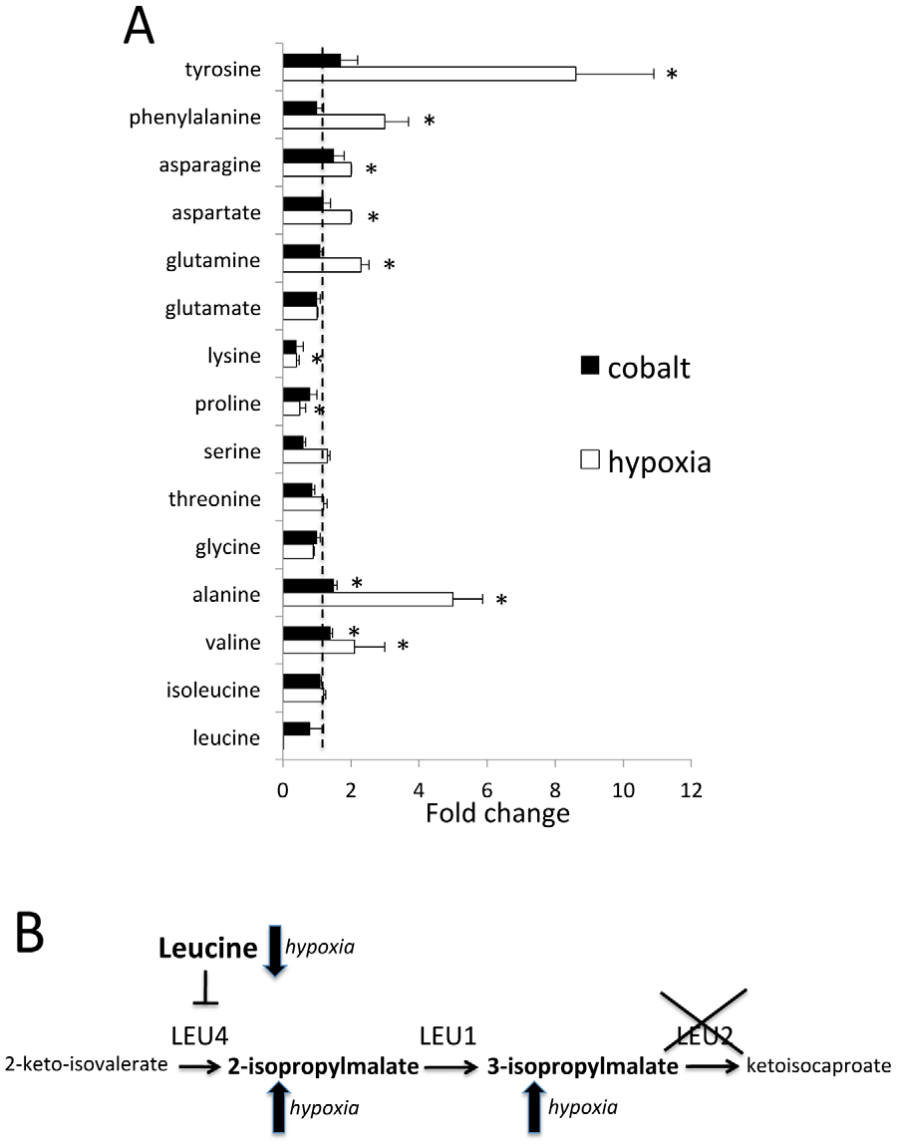
Effect of hypoxia on amino acids. A) Cells grown in minimal medium as in Fig. 1 were assayed for the designed amino acids by GC/MS. Statistically significant differences over control (P value  = 0.05) are designated with asterisks. Dotted line  = 1.0 value assigned to control. B) Segment of the leucine biosynthetic pathway involving Leu4p, Leu1p (IPMI) and Leu2p. The strain used in these studies contains a *leu2* gene deletion designated by X. Arrows indicate the effect of hypoxia on leucine and the 2-IPM and 3-IPM precursors for leucine. Schematic depicts the negative feedback of leucine levels on Leu4p.

Unlike cobalt, the most notable effect of hypoxia was on the 2-IPM and 3-IPM substrate and product for IPMI, which were increased by nearly 50 fold upon oxygen deprivation (Fig. 7A). In spite of these changes, hypoxia did not substantially alter the polypeptide levels nor enzymatic activity of IPMI (Fig. 7B,C). Our additional studies (see ahead Fig. 8) indicate that the rise in 2-IPM and 3-IPM with hypoxia reflects increased flux through the leucine biosynthetic pathway.

### Hypoxia specific effects on amino acid pools

**Figure 9:**
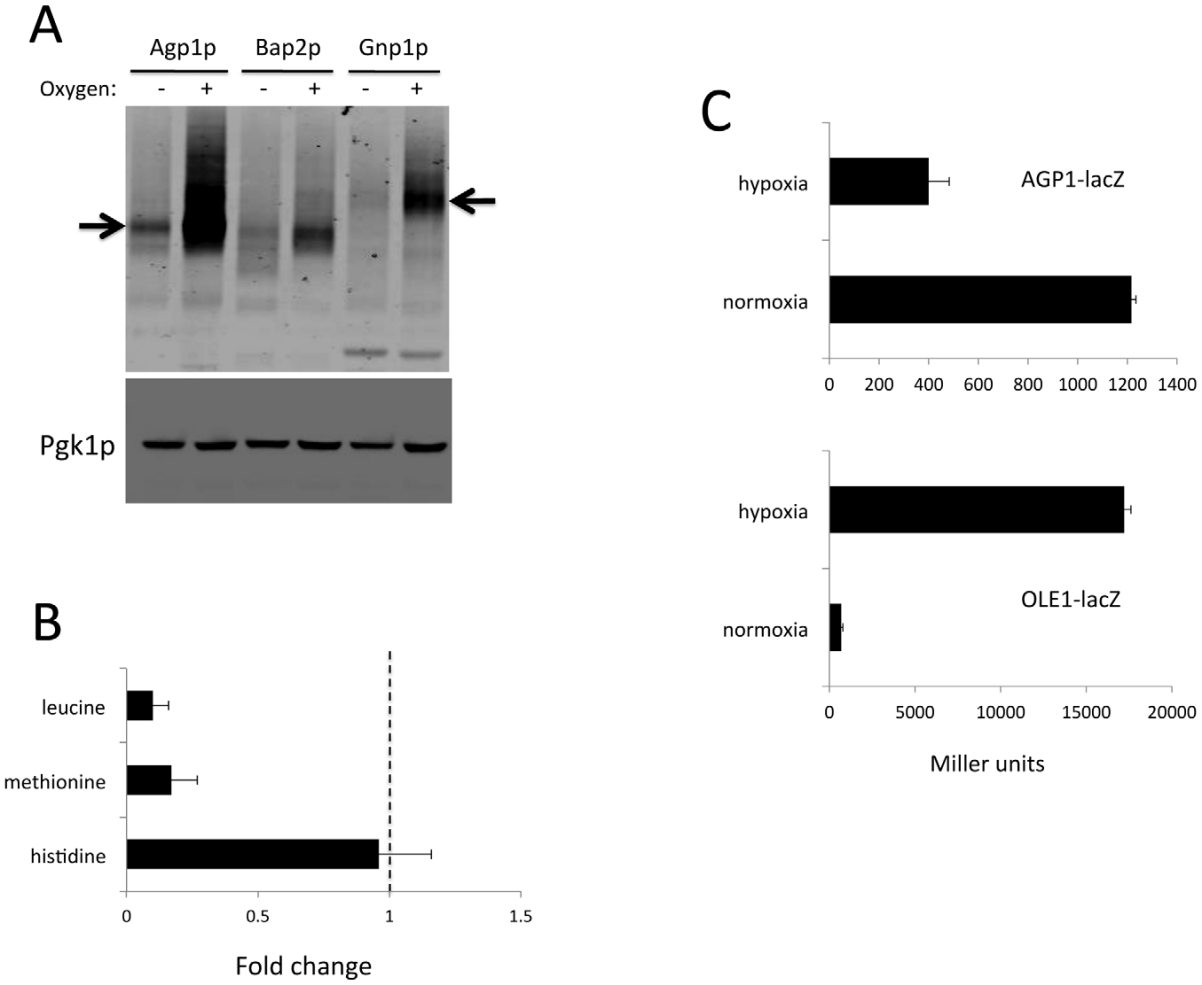
Hypoxia down-regulates amino acid transporters. Cells grown in minimal medium as in Fig. 1 were assayed for expression of amino acid transporters (A), select amino acids (B) and *lacZ* reporter activities (C). A) Immunoblot analysis of cells expressing TAP tagged versions of Agp1p (90.7 kDa), Bap2p (88.1 kDa) and Gnp1 (94,6 kDa). Arrows indicate positions of Agp1p and Gnp1p. Results are representative of four experimental trials. B) Levels of the indicated amino acids were measured from triplicate cell samples by ion exchange chromatography. Error bars represent standard deviation. Fold change represents values from hypoxia grown samples over that of control aerobic cells. C) Cells transformed with plasmids for expressing *lacZ* under the control of the *AGP1* (top) or *OLE1* (bottom) promoter were assayed for ß-galactosidase as in Materials in Methods. Results represent the averages of 2–3 independent transformants and error bars represent range. Values shown are in Miller units.

In the course of our studies, we noted a striking effect of hypoxia on amino acid pools that were not seen with cobalt (Table S1 and Fig. 8A). For example, the aromatic amino acids tyrosine and phenylalanine consistently increased with hypoxia, as did alanine and valine. Most notably, leucine levels dropped to concentrations that ranged from low (Fig. 9A) to virtually undetectable (Fig. 8A and Table S1) in various experimental trials. This drop in leucine may very well explain the dramatic elevations in the 2-IPM and 3-IPM precursors (Fig. 7A). Leucine is a negative feedback regulator of Leu4p, the IMP synthase depicted in Fig. 8B [27]. With hypoxia, such low leucine levels should lead to activation of Leu4p and hyperaccumulation of 2-IPM and 3-IPM, particularly in this strain background where *LEU2* is disrupted (see Fig. 8B).

Because of the *leu2* mutation, leucine is a required amino acid for the strain of yeast used in these studies and needs to be taken up from the growth medium. Transporters for leucine include the closely related permeases Agp1p, Bap2p and Gnp1p [28]. As seen in Fig. 9A, all three transporters are dramatically decreased with hypoxia, consistent with the observed drop in cellular leucine. Methionine and histidine are also required amino acids due to *met15* and *his3* gene deletions. Methionine and histidine were missing from the original GC/MS profile (Fig. 8A and Table S1); therefore a more detailed amino acid analysis was carried out using ion exchange chromatography. As seen in Fig. 9B, methionine levels also dropped with hypoxia; however, histidine was unchanged. It is noteworthy that like leucine, methionine is taken up by the aforementioned Agp1p, Bap2p and Gnp1p transporters, whereas histidine is transported by the Hip1p histidine specific permease [28].

We investigated the mechanism for hypoxia down regulation of amino acid permeases. These transporters can be controlled at the level of protein stability through degradation at the cell surface or in the secretory pathway [29], [30]. However, hypoxia did not induce degradation of Agp1p; the stability of the protein in air and hypoxia were comparable (Fig. S2). The permeases are also controlled at the level of transcription in response to extracellular levels of amino acids. The genes for Agp1p, Bap2p and Gnp1p (but not the histidine permease Hip1p) are induced through an amino acid sensing and signaling pathway known as SPS [31]. Extracellular amino acids, particularly leucine, react with a plasma membrane SPS-sensor that activates Stp1p and Stp2p transcription factors for amino acid permease genes [31]. In all our studies, leucine is present in the growth medium and the SPS pathway should be active. To test whether hypoxia interferes with this pathway, we employed a *AGP1-lacZ* reporter that is used to monitor the SPS response [32]. As seen in Fig. 9C, *AGP1-lacZ* reporter activity decreased by approximately three fold with hypoxia. This loss of SPS reporter activity was not due to inhibition of ß-galactosidase activity under our hypoxic conditions, as an *OLE1-lacZ* reporter examined in parallel exhibited strong induction in hypoxia (Fig. 9C). These studies provide evidence for the first time that hypoxia can interfere with signaling for amino acid uptake.

## Discussion

The metabolite profiling approach described here provides new insight into the overlapping and distinct impacts of hypoxia and a “hypoxia-like” agent cobalt on cell metabolism. In *S. cerevisiae,* cobalt can partially simulate hypoxia by depleting cellular sterols and unsaturated fatty acids and this involves cobalt-mediated inhibition of biosynthetic steps involving diiron-oxo enzymes. The concomitant lowering of key sterols and/or changing the UFA/SFA ratio is likely to trigger the hypoxia-like transcriptional response that has been reported for cobalt [13], [14], [15]. It is possible that other diiron enzymes in *S. cerevisiae* are affected, e.g., ribonucleotide reductase (RNR), although with the doses of cobalt used herein, we did not observe a cell cycle defect as would be expected for loss of RNR (Fig. S3). Diiron enzymes that require oxygen are distributed widely in evolution, for example mammals use diiron-oxo desaturases for UFA synthesis and a diiron-oxo hydroxylase for sterol metabolism [24], [33]. Hence, the hypoxia-like properties of cobalt in higher organisms are likely to include effects on diiron-oxo enzymes as well. In spite of these common effects on sterols and fatty acids, metabolite profiling studies have pinpointed divergent impacts of cobalt and low oxygen on specific Fe-S enzymes and amino acid pools.

Of the various Fe-S enzymes, cobalt appeared to preferentially target mitochondrial 4Fe-4S dehydratases (e.g., aconitase and homoaconitase), while the homologous 4Fe-4S IPMI in the cytosol was spared from cobalt. The attack on mitochondrial Fe-S enzymes by cobalt may very well explain the reported cobalt induction of the Aft1p iron starvation response, which is known to be activated by loss of mitochondrial 4Fe-4S clusters [16], [18], [19]. But why is cytosolic 4Fe-4S IPMI not affected by cobalt? It is possible that cobalt is more bioavailable in the mitochondria compared to the cytosol. Alternatively, the differential effects on mitochondrial versus cytosolic Fe-S dehydratases could reflect lability of the corresponding 4Fe-4S cluster *in vivo*. The 4Fe-4S dehydratases contain a particularly labile cluster [26], and in the oxidizing environment of the mitochondria, the Fe-S groups from aconitase like enzymes should be more prone to damage and require more rapid assembly and repair than their counterparts (IPMI) in the cytosol. Therefore, cobalt may interfere with the assembly or repair of those mitochondrial Fe-S clusters subject to rapid turnover. A similar scenario has been used to explain cobalt damage of Fe-S enzymes in *Salmonella* and *E. coli* [34], [35], [36], [37]. In any case, Fe-S clusters can be added to the growing list of iron containing enzymes that are disrupted by cobalt, including the non-heme iron 2-oxoglutarate enzymes (hydroxylases for HIF-1α and the JmjC domain histone demethylases; [9], [10], [38]) and the diiron-oxo desaturases (Erg3p, Erg25p and Ole1p) described here. It is noteworthy that not all classes of iron enzymes are prone to cobalt attack, as we obtained no evidence for loss of heme containing enzymatic steps.

Recently, Philpott and colleagues conducted a comprehensive metabolomic screen on yeast cells starved for iron [39]. Surprisingly, there was minimal overlap noted between iron starvation and cobalt. Iron starvation did inhibit the mitochondrial Fe-S aconitase and homoaconitase but unlike cobalt, iron starvation also impacted cytosolic IPMI/Leu1p. Heme containing enzymes in sterol biosynthesis were also affected by low iron but there was no evidence for loss of the diiron-oxo Ole1p enzyme as we show here for cobalt. It was suggested that yeast adaptation to low iron sufficiently spares critical iron-proteins like Ole1p [39], but there may be no paralleled sparing of essential iron enzymes with cobalt.

One of the most remarkable outcomes of this metabolomic study was the effect of low oxygen on amino acids in yeast. For example, amino acids such as alanine and the aromatics phenylalanine and tyrosine consistently increased with hypoxia. In a recent metabolomic study using piglets, a number of amino acids increased with asphyxia, most notably alanine [40]. The elevation in such amino acids during low oxygen could involve increased biosynthesis of amino acids, increased protein turnover or decreased translation. In *S. cerevisiae,* the *ARO3* and *ARO4* genes for aromatic amino acid biosynthesis are induced by amino acid starvation [41], but we noted that Aro3p and Aro4p levels do not change with hypoxia (Fig. S4), suggesting that increased amino acid synthesis is not the cause. Hypoxia can suppress translation in *C. elegans* [42], and increases in protein turnover as occurs in autophagy during stress could also account for increased amino acid pools with hypoxia [43], [44]. In any event our studies together with the recent metabolomic studies on piglets [40] indicate that increases in specific amino acids can be novel indicators of metabolic stress induced by hypoxia.

Compared to effects on the aromatic amino acids and alanine, leucine and methionine levels dramatically decreased with yeast hypoxia due to loss of cell surface permeases for these amino acids. In *S. cerevisiae*, extracellular amino acids, particularly leucine, activate a SPS response in which the permease genes are induced through Spt1p and Spt2p transcription factors [32], [45]. In our studies, there was sufficient leucine in the growth medium to activate this SPS response and the permease genes were well induced in aerobic conditions, but not in hypoxia. There are multiple possible targets where hypoxia might interfere with this signal, including inhibition of amino acid sensing by the cell surface SPS receptor, inhibition of processing of the Spt1p/Spt2p trans-regulators or its transcriptional activity within the nucleus [31], [46]. Alternatively, hypoxia may be acting on an unknown factor for regulation of amino acid permease genes. Why should amino acid uptake be blocked by hypoxia? The Agp1p, Gnp1p and Bap2p permeases are relatively non-specific and it may be advantageous to block amino acid uptake when intracellular amino acid pools are rising. In any case, our studies are the first to report hypoxia control of amino acid pools and it will be intriguing to define the new molecular target(s) of hypoxia in this regard.

In conclusion, these studies illustrate the power of metabolomics for defining new pathways and targets of cell stressors. For example, the rise in metabolite substrates for mitochondrial, but not cytosolic, Fe-S dehydratase enzymes that we observed with cobalt completely correlated with inhibition of the corresponding enzymatic activity without changes in dehydratase protein levels. Hence, metabolite profiling can reveal immediate targets of toxins in the absence of any changes in RNA or protein levels. Of course there are limitations to this approach, as not all losses in enzymatic activity will be revealed by changes in corresponding substrates or products. Nevertheless metabolomics can help pinpoint new pathways for further exploration by more molecular means, as is the case for our newly described impact of hypoxia on amino acid pools.

## Materials and Methods

### Yeast strains and culture conditions

The yeast strains used for these studies were all derived from BY4741 (*MatA, his3Δ1, leu2Δ0, met15Δ0, ura3Δ0*). Immunoblot analyses employed derivatives of BY4741 that harbored C-terminally TAP-tagged versions of specified proteins (Open Biosystems) [47]. For ß-galactosidase analysis of *APG1* and *OLE1* promoter activity, BY4741 was transformed with the YCpAGP1-lacZ [32] and pAM6 (kind gift of C. Martin [14]) respectively. Cells were grown in either an enriched yeast extract peptone based medium (YPD) or in a minimal medium (SD) supplemented per liter with 20 g glucose; SD medium also contained 60 mgs leucine and 20 mgs each of histidine, methionine and uracil per liter [48].

For studies in minimal medium including metabolite profiling, immunoblots and enzymatic assays for aconitase, IPMI and ß-galactosidase, cells from a stationary phase aerobic culture were harvested and resuspended to an OD_600_ of 0.125 in10 mls of SD medium and allowed to grow for precisely 3 doublings (to OD_600_ of 1.0) under the following three conditions: air untreated; air in medium supplemented with 2 mM cobalt, a concentration that inhibits 24 hour growth by 50%; under hypoxic conditions without cobalt. For the latter, cells were transferred to a Coy Laboratory anaerobic workstation equilibrated to 30°C and resuspended in SD medium that had been stored in the same anaerobic environment. In all three cases, cells were grown by shaking at 200 rpm in 25×150 mm borosilicate glass tubes that each contained approximately four 3 mM glass beads to prevent cell coagulation. Following growth, cells were harvested and washed once in water prior to flash freezing and biochemical analyses.

### GC-MS analysis of diverse metabolites

Eight replicate cell samples were extracted by the addition of 5 ml of boiling 75% EtOH (aqueous) followed by incubation at 90°C for five minutes. Cell debris was removed by centrifugation at 5000× g for three minutes. The isolated supernatant was dried *en vacuo*. GC-MS analysis was performed using a GCT Premier (Waters) mass spectrometer fitted with an Agilent 6890 gas chromatograph. A Gerstel MPS2 autosampler was employed in conjunction with a CIS4 cold injection inlet. Dried samples were suspended in 40 µl of a 40 mg/mL O-methoxylamine hydrochloride in pyridine and incubated for one hour at 30°C. 25 ml of this solution was mixed with 25 µl of MSTFA (N-methyl-N-trimethylsilyltrifluoroacetamide) followed by incubation for 30 minutes at 37°C with shaking. 1 µl of this sample was injected to the cool inlet with the following program, 75°C for 30 seconds followed by a 10°C/second ramp to 250°C and a hold time of 2 minutes. The gas chromatograph had an initial temperature of 75°C for one minute followed by a 40°C/minute ramp to 110°C and a hold time of 2 minutes. This was followed by a second 5°C/minute ramp to 250°C then a third ramp to 350°C with a final hold time of 3 minutes. A 30 m Restek Rxi-5 MS column with a 5 m long guard column was employed for analysis. Data was collected by MassLynx 4.1 (Waters, Beverly MA). Primary analysis was performed by QuanLynx. Further analysis was performed using MarkerLynx and this data was exported to SIMCA-P ver 12.0 (Umetrics, Kinnelon, NJ) where principle component analysis and partial least squares-discriminate analysis (PLS-DA) was performed. Chromatographic features which contributed to the PLS-DA model were analyzed in further detail with each peak being tested for significance. This GC-MS analysis was used for sterols (Figs. 1–2), Fe-S enzyme metabolites (Fig. 7) and amino acids (Fig. 8).

### GC analysis of Fatty acids and sterols

For analysis of fatty acids, 10 OD_600__nm_ units of cells that were either grown in SD medium as described above or in YPD medium to an OD_600__nm_  = 1.0 were harvested and resuspended in 1 ml cold methanol and transferred to a glass tube where an additional 1 ml methanol, 1 ml chloroform and 0.8 mls 1 M NaCl were added. Samples were mixed by vortexing and incubated at room temperature for 30 min prior to adding 1 ml each of chloroform and 1.0 M NaCl. Fatty acids were extracted by vortexing and centrifugation and by removing the chloroform containing layer, followed by a back extraction with 2 mls chloroform. The two chloroform layers were combined, dried under nitrogen and resuspended in 250 µl chloroform followed by a second round of drying. Samples were resuspended in 150 µl methanolic sulfuric acid (98% methanol, 2% sulfuric acid), briefly purged with nitrogen, followed by heat treatment at 70°C for 2 hours and brief cooling at room temperature. Samples were resuspended in 75 µl water and extracted with 150 µl pentane. The top pentane containing layer was removed, and the aqueous phase re-extracted with another 150 µl pentane. The pentane layers were combined, dried under nitrogen, and the fatty acid residue suspended in 500 µl hexane prior to analysis by gas chromatography.

For analysis of sterols, cells grown in SD or YPD medium were resuspended in 0.2 mls water and were mixed with 9 mL methanol and 4.5 mL 60% KOH. 5 µg cholesterol was added as a recovery standard. Samples were vortexed and incubated at 75°C for 2 hours, cooled at room temp for 15 minutes followed by extraction with 4 mls petroleum ether. The top phase was removed, dried under nitrogen and the sterol containing residue dissolved in 500 µl heptane prior to analysis by gas chromatography as previously described [12]. Such GC analysis was used for measurements of squalene in SD grown cells (Fig. 1) and for analysis of squalene, lanosterol, ergosterol, zymosterol, episterol, 5,7,22,24(28) tetraenol and 4-methyl fecosterol of YPD grown cells as was previously done [12]. (Fig. S1).

### Amino acid analysis by ion exchange chromatography

Triplicate cell samples, each comprising 40 OD_600_ units of cells (obtained by combining four 10 ml cultures as described above), were harvested and washed in PBS (phosphate buffered saline). Samples were suspended in 0.5 ml PBS, combined with zirconium oxide beads and lysed by glass bead homogenization in a TissueLyser (10 minutes at 50 Hz). Cell lysates were then clarified by centrifugation at 17,000xg and the protein concentration determined by Bradford analysis. Sulfosalicylic acid was added to a final concentration of 3.2% and incubated for 5 minutes at room temperature to precipitate proteins. Samples in triplicate were then subjected to two successive rounds of centrifugations for 3 minutes at 17,000xg to collect soluble amino acids. Amino acid analysis was then conducted by standard ion exchange chromatography procedures using a Biochrom 30 amino acid analyzer at the Kennedy Krieger Institute Biochemical Genetics Laboratory [49]. Ion exchange chromatography was used in the analysis of amino acids for Fig. 9.

### Metal toxicity and accumulation analyses, immunoblots and enzymatic assays

For metal toxicity tests and atomic absorption analysis of cobalt, cells were seeded in YPD medium at OD_600__nm_  = 0.05 and grown at 30°C for 18 hours in the designated concentrations of CoCl_2_, ZnCl_2_, MnCl_2_ or NiCl_2_. With metal analysis, duplicate cultures were washed and prepared for atomic absorption spectroscopy as previously described [50]. For immunoblots, 10 OD_600_ units of cells grown either in SD medium or in YPD overnight to confluency were washed and resuspended in 250 µl buffer containing 10 mM sodium phosphate pH 7.8, 0.1% Triton X-100, 5 mM EDTA, 5 mM EGTA, 50 mM NaCl. Cells were lysed in a TissueLyser using zirconium oxide beads by vortexing twice for two minutes at 50 Hz. Lysates were subject to 12% PAGE and immunoblotting using anti-TAP antibody (1∶5000 dilution; Open Biosystems) or anti-PGK1 (1∶5000 dilution; Invitrogen).

Aconitase (Aco1p) and isopropylmalate isomerase or IPMI (Leu1p) activity assays were conducted on SD grown cells. Briefly, 5×10^8^ cells suspended in 0.2 ml of deoxygenated lysis buffer (10 mM NaPO4 (pH 7.6), 0.1 mM EDTA, 0.1 mM EGTA, 0.1% Triton X-100, 50 mM NaCl, 1.0 mM phenylmethylsulfonyl fluoride) were subjected to glass bead lysis under a nitrogen atmosphere in a COY chamber. Aco1p and Leu1p activity was determined spectrophotometrically using a BioTek Synergy HT plate reader. The assay mixture contained 50–200 µg of lysate protein in 200 µl of a buffer containing 50 mM tris(hydroxymethyl)aminomethane (Tris)-HCl, pH 7.4 and100 mM NaCl and supplemented with either 0.5 mM cis-aconitate (Aco1p activity) or 0.5 mM citraconitate (Leu1p activity). Activities were determined by monitoring the disappearance of cis-aconitate (Aco1p) or citraconitate (Leu1p) at 240 nm or 235 nm, respectively, over the course of 3 minutes. These species were quantified by generating calibration curves of standardized concentrations of cis-aconitate or citraconitate. In both cases, 1 unit of activity is defined as 1 nmole of substrate consumed per min per mg of protein.

β-galactosidase activity was assayed as previously described [51] using *ortho*-nitrophenyl-β-d-galactopyroanoside (ONPG) as a substrate in 96 well plates. The absorbance of the samples at 420 nm was monitored kinetically for 30 minutes at 30°C using a BioTek Synergy HT plate reader. Results are reported in Miller units [52]. Duplicate or triplicate transformants were assayed in duplicate.

## Supporting Information

Figure S1 **Cobalt effects on the ergosterol pathway in cells grown in enriched YPD medium.** Cells were grown in enriched YPD medium in the presence of the indicated concentrations of CoCl_2_ and were subject to sterol metabolite analysis by GC as described in Materials and Methods. (A,B) Results represent fold changes over control  =  cells grown without cobalt. C) Episterol and 4- methyl fecosterol (4-MF) were only detected in cobalt treated cells and thus the arbitrary units shown represent the quantity of metabolite detected above control samples which  = 0, precisely as was done for Fig. 2A. Results represent averages of two independent cultures (error bars  =  standard deviation) and are representative of two experimental trials. As was seen with GC MS analysis of SC grown cells (Fig. 1), cobalt treatment of YPD grown cells results in an increase of upstream metabolites of the ergosterol pathway (squalene and lanosterol, part A), and a decrease in downstream products (zymosterol, 5,7,22,24(28) tetraenol and ergosterol, part B). In addition, cobalt treatment in YPD results in abundant accumulation of 4 methyl fecosterol (4-MF, part C), a marker of Erg25p inactivation, precisely as was seen with SD grown cells (Fig. 2A). In YPD grown cells, the episterol substrate for Erg3p could not be detected without cobalt, but was seen to accumulate with cobalt treatment (part C), consistent with Erg3p inactivation.
(PDF)

Figure S2 **Hypoxia does not result in increased turnover of Agp1 as determined by protein inhibition with cycloheximide.** Cells expressing TAP tagged versions of Agp1p were grown in minimal medium and subject to protein turnover studies by immunoblot analysis (as in Fig. 9A) in two ways: (A) cells were allowed to double twice to an OD_600_ = 0.5 either in air or under hypoxia conditions prior to the addition of 100 µg/ml cycloheximide for the indicated time in hours. The lower level of starting material (+chx: 0) with hypoxia reflects hypoxia repression of Agp1 synthesis prior to chx treatment. (B) Equal starting material for hypoxia and aerobic conditions was used and represented cells grown aerobically to OD_600_ = 0.5. Cells were harvested and resuspended in medium that contained 100 µg/ml cycloheximide and preconditioned for hypoxia where indicated, followed by incubation at 30^o^C for the indicated times under either hypoxic or aerobic conditions. 100 µg/ml cycloheximide is typically used for protein synthesis inhibition studies in *S. cerevisiae* and indeed inhibited translation in these experiments as indicated by complete and immediate cessation of growth and decreased protein recovery in lysates. Results are representative of 2 experimental trials.
(PDF)

Figure S3 **Cobalt treatment does not result in an apparent cell cycle defect.** Cells were grown in SD medium in the presence or absence of cobalt precisely as was for analysis of sterols (Figs. 1B, 2A) and fatty acids (Fig. 4B). Cells were stained with DAPI to view nuclear and mitochondrial DNA and subject to fluorescence microscopy using a 100X objective Zeiss Observer.Z1 microscope. Images were obtained using the Zeiss Axiovision sofware. Shown are the overlay of DAPI fluorescence (blue) and DIC light microscopy images of whole cells. The characteristic large budded cells typical of the cell cycle defects associated with loss of ribonucleotide reductase [53] were not seen with these cobalt treated cells.
(PDF)

Figure S4 **Hypoxia does not induce levels of Aro3p and Aro4p for biosynthesis of phenyalanine and tyrosine.** Cells expressing TAP tagged versions of the indicated proteins were grown in minimal medium as in Fig. 1 and analyzed by immunoblot using an antibody directed against TAP. Shown are expression levels of TAP tagged-Aro3p (61 kDa) and Aro4p (62 kDa). Results are representative of three individual experimental trials.
(PDF)

Table S1 **Metabolite profiles for yeast grown in the absence of oxygen or the presence of cobalt.** Cells were grown and prepared for analysis by GC/MS as described in Materials and Methods. Shown are the composite list of metabolites analyzed. In two independent experimental trials, the fold change of 8 individual treated (hypoxia or cobalt) over 8 individual control untreated was calculated, with P values as determined in a T-test. The means calculated over the two experimental trials with standard deviation is shown. Numbers in black reflect statistically significant values where the P values for both experimental trials were <0.05.
(XLS)
