# Supplementary material for: Analysis of Hypoxia and Hypoxia-Like States through Metabolite Profiling
Source: PLoS One. 2011 Sep 12;6(9):e24741. doi: 10.1371/journal.pone.0024741 (PMC3171472; doi:10.1371/journal.pone.0024741)

Supplementary Figure 1: Cobalt effects on the ergosterol pathway in cells grown in enriched YPD medium

A

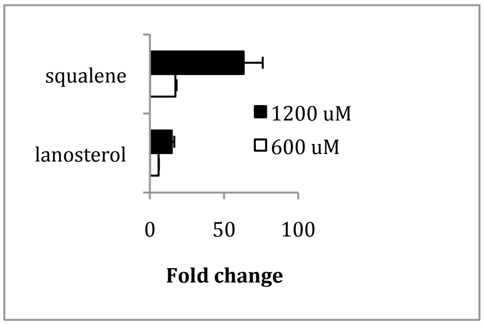

B

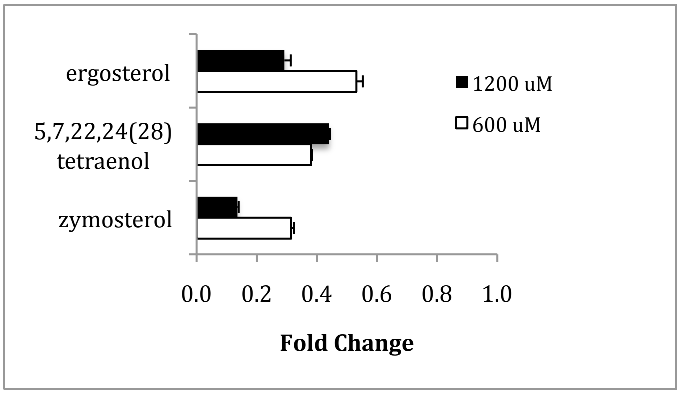

C

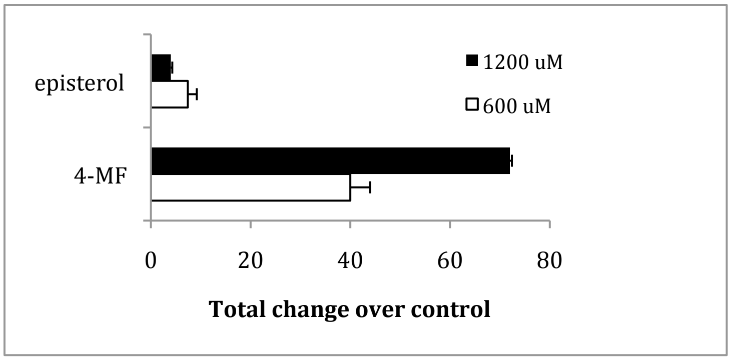

Supplement: Figure S1 — Cobalt effects on the ergosterol pathway in cells grown in enriched YPD medium. Cells were grown in enriched YPD medium in the presence of the indicated concentrations of CoCl2 and were subject to sterol metabolite analysis by GC as described in Materials and Methods. (A,B) Results represent fold changes over control = cells grown without cobalt. C) Episterol and 4- methyl fecosterol (4-MF) were only detected in cobalt treated cells and thus the arbitrary units shown represent the quantity of metabolite detected above control samples which = 0, precisely as was done for Fig. 2A. Results represent averages of two independent cultures (error bars = standard deviation) and are representative of two experimental trials. As was seen with GC MS analysis of SC grown cells (Fig. 1), cobalt treatment of YPD grown cells results in an increase of upstream metabolites of the ergosterol pathway (squalene and lanosterol, part A), and a decrease in downstream products (zymosterol, 5,7,22,24(28) tetraenol and ergosterol, part B). In addition, cobalt treatment in YPD results in abundant accumulation of 4 methyl fecosterol (4-MF, part C), a marker of Erg25p inactivation, precisely as was seen with SD grown cells (Fig. 2A). In YPD grown cells, the episterol substrate for Erg3p could not be detected without cobalt, but was seen to accumulate with cobalt treatment (part C), consistent with Erg3p inactivation. (PDF) [file pone.0024741.s001.pdf]
