# Supplementary material for: Analysis of Hypoxia and Hypoxia-Like States through Metabolite Profiling
Source: PLoS One. 2011 Sep 12;6(9):e24741. doi: 10.1371/journal.pone.0024741 (PMC3171472; doi:10.1371/journal.pone.0024741)

Supplementary Figure 2:
Hypoxia does not result in increased turnover of Agp1 as determined by protein inhibition with cycloheximide

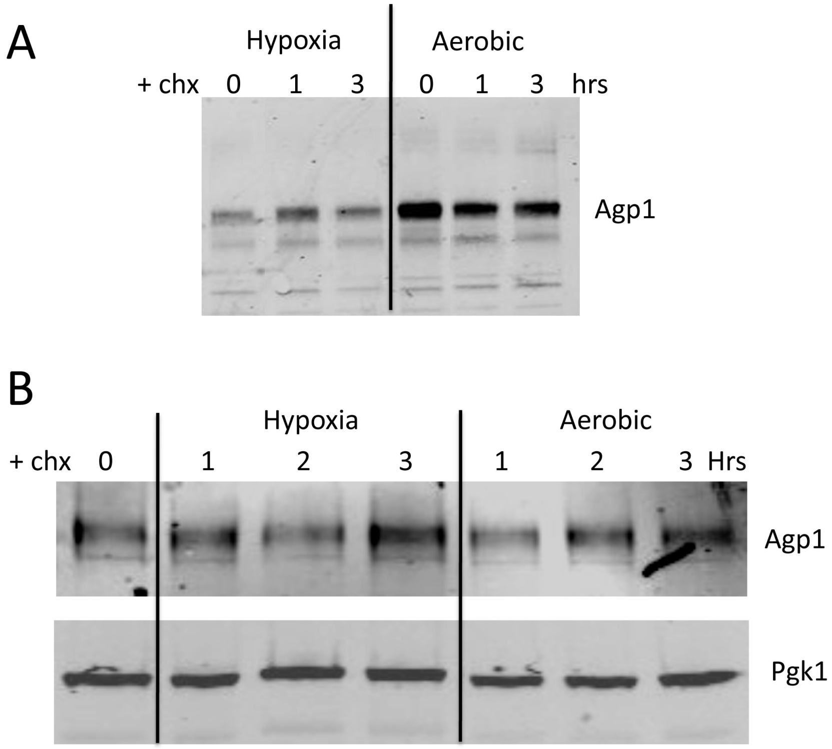

Supplement: Figure S2 — Hypoxia does not result in increased turnover of Agp1 as determined by protein inhibition with cycloheximide. Cells expressing TAP tagged versions of Agp1p were grown in minimal medium and subject to protein turnover studies by immunoblot analysis (as in Fig. 9A) in two ways: (A) cells were allowed to double twice to an OD600 = 0.5 either in air or under hypoxia conditions prior to the addition of 100 µg/ml cycloheximide for the indicated time in hours. The lower level of starting material (+chx: 0) with hypoxia reflects hypoxia repression of Agp1 synthesis prior to chx treatment. (B) Equal starting material for hypoxia and aerobic conditions was used and represented cells grown aerobically to OD600 = 0.5. Cells were harvested and resuspended in medium that contained 100 µg/ml cycloheximide and preconditioned for hypoxia where indicated, followed by incubation at 30oC for the indicated times under either hypoxic or aerobic conditions. 100 µg/ml cycloheximide is typically used for protein synthesis inhibition studies in S. cerevisiae and indeed inhibited translation in these experiments as indicated by complete and immediate cessation of growth and decreased protein recovery in lysates. Results are representative of 2 experimental trials. (PDF) [file pone.0024741.s002.pdf]
