# Supplementary figures and images for: Analysis of Hypoxia and Hypoxia-Like States through Metabolite Profiling
Source: PLoS One. 2011 Sep 12;6(9):e24741. doi: 10.1371/journal.pone.0024741 (PMC3171472; doi:10.1371/journal.pone.0024741)

Supplementary Figure 3: Cobalt treatment does not result in an apparent cell cycle defect.

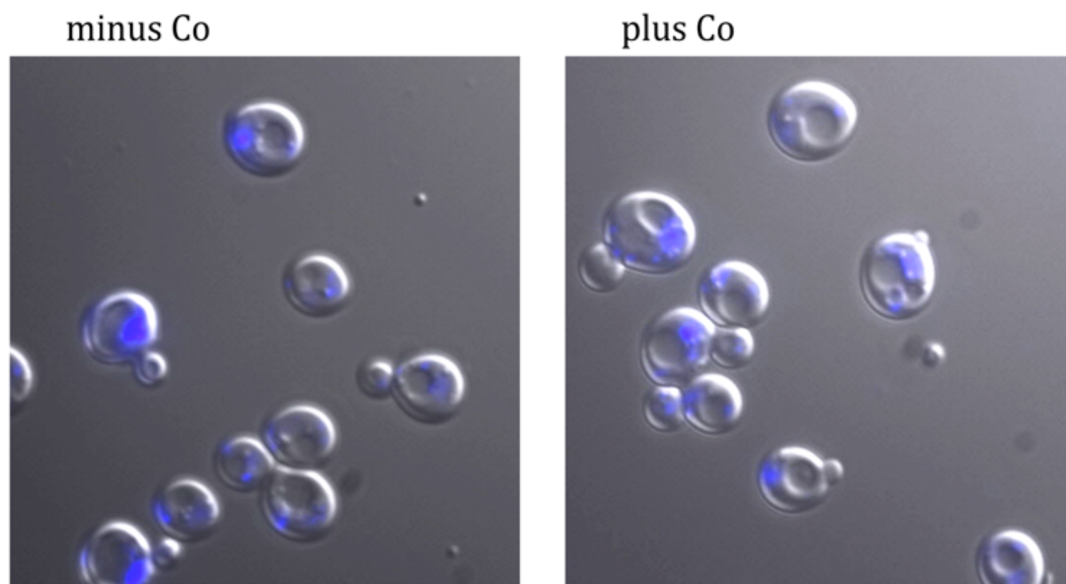

Supplement: Figure S3 — Cobalt treatment does not result in an apparent cell cycle defect. Cells were grown in SD medium in the presence or absence of cobalt precisely as was for analysis of sterols (Figs. 1B, 2A) and fatty acids (Fig. 4B). Cells were stained with DAPI to view nuclear and mitochondrial DNA and subject to fluorescence microscopy using a 100X objective Zeiss Observer.Z1 microscope. Images were obtained using the Zeiss Axiovision sofware. Shown are the overlay of DAPI fluorescence (blue) and DIC light microscopy images of whole cells. The characteristic large budded cells typical of the cell cycle defects associated with loss of ribonucleotide reductase [53] were not seen with these cobalt treated cells. (PDF) [file pone.0024741.s003.pdf]
