# Supplementary material for: Analysis of Hypoxia and Hypoxia-Like States through Metabolite Profiling
Source: PLoS One. 2011 Sep 12;6(9):e24741. doi: 10.1371/journal.pone.0024741 (PMC3171472; doi:10.1371/journal.pone.0024741)

Supplementary Figure 4:
Hypoxia does not induce levels of Aro3p and Aro4p for biosynthesis of phenylalanine and tyrosine

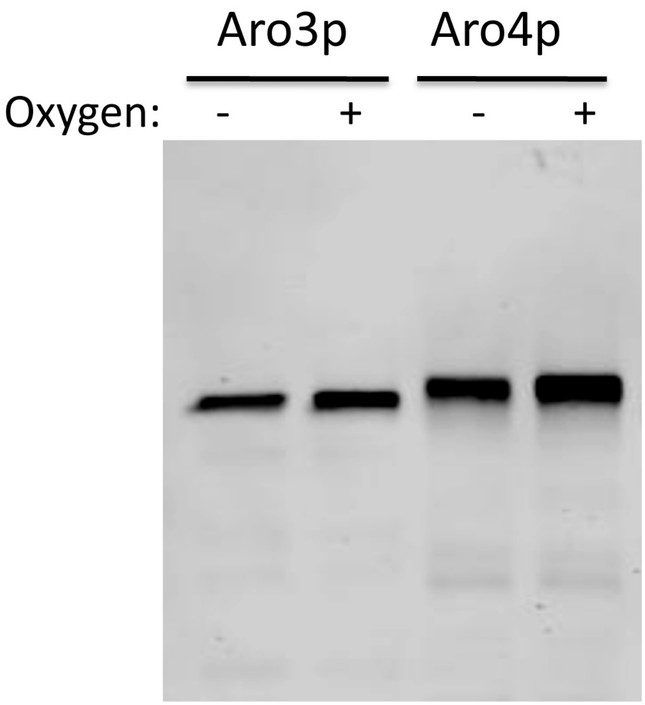

Supplement: Figure S4 — Hypoxia does not induce levels of Aro3p and Aro4p for biosynthesis of phenyalanine and tyrosine. Cells expressing TAP tagged versions of the indicated proteins were grown in minimal medium as in Fig. 1 and analyzed by immunoblot using an antibody directed against TAP. Shown are expression levels of TAP tagged-Aro3p (61 kDa) and Aro4p (62 kDa). Results are representative of three individual experimental trials. (PDF) [file pone.0024741.s004.pdf]
